# Cervical Tuberculous Lymphadenitis

**DOI:** 10.7759/cureus.31282

**Published:** 2022-11-09

**Authors:** Luis F Lemus, Ernesto Revelo

**Affiliations:** 1 School of Medicine, Universidad Dr. José Matías Delgado, San Salvador, SLV; 2 Internal Medicine, Hospital San Rafael, Santa Tecla, SLV

**Keywords:** extrapulmonary tuberculosis (eptb), mycobacterium tuberculous, neck mass, caseous necrosis, lymph node tuberculosis

## Abstract

Neck masses are a very common clinical problem and it remains a diagnostic challenge due to multiple differential diagnoses ranging from benign to severe etiologies. All physicians should equip themselves with knowledge of common and locally prevalent causes of neck masses and manage them accordingly. We present a case of a young patient with no prior medical history who developed cervical tuberculous lymphadenitis. We discuss the physical examination, evolution, diagnosis, and treatment of the case.

## Introduction

Tuberculosis (TB) is an infectious disease caused by *Mycobacterium tuberculosis* which mainly affects the lungs, making pulmonary disease the most common presentation. However, TB is a multi-systemic disease that may affect many organs and can present without symptoms as well. It may affect extrapulmonary sites such as the gastrointestinal system, lymph nodes, skin, central nervous system, musculoskeletal system, reproductive system, liver, and spleen [1]. 

TB is a preventable and treatable disease but it is still one of the major contributors to morbidity and mortality in developing countries. Global measures are still being taken to control the spread of this disease [1]. In El Salvador, the disease burden is significantly high. 

Here, we present a case report that highlights an unusual presentation of tuberculosis in our country. We discuss the clinical presentation of the patient, diagnostic studies, and treatment based on our local guidelines for the management of this disease.

## Case presentation

**Figure 1:**
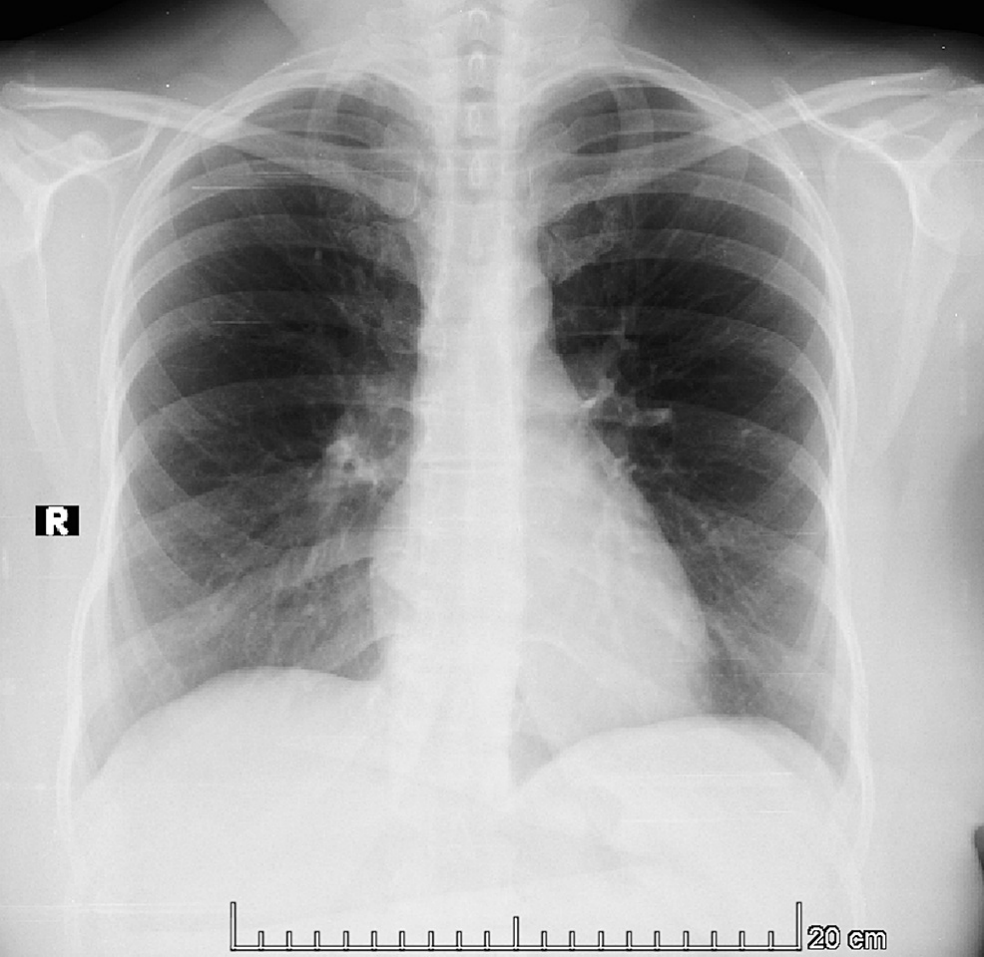
PA X-ray of the chest showed no consolidations, no infiltrations, and no granulomas at the apex.

**Figure 2:**
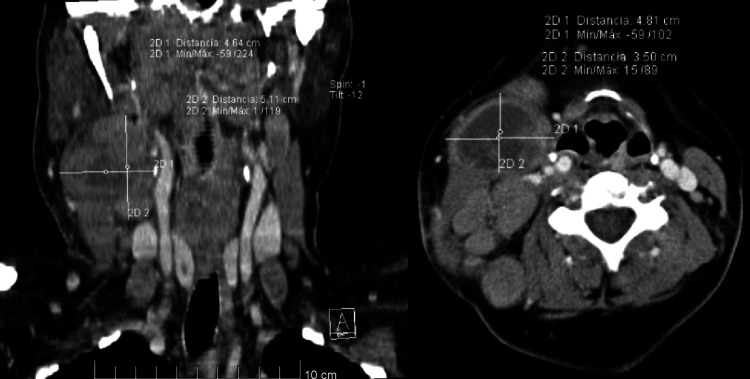
CT scan of the neck showed a mass with no compression of nervous or vascular structures.

A 46-year-old female from El Salvador presented to the emergency department of San Rafael National Hospital with a history of non-painful swelling in the right side of the neck that had been gradually increasing in size for the past four months. There was no history of fever, night sweats, respiratory symptoms, gastrointestinal symptoms, or weight loss. The patient had no significant past medical or surgical history and there was no family history of cancer and no contact with patients with tuberculosis. At the time the mass measured around 5x5 cm smooth to palpation, painless, not fixed to tissue with no local signs of inflammation. Ultrasound of the neck showed multiple infiltrative adenopathies with loss of morphology measuring between 9.0 mm to 32.0 mm. The patient underwent a biopsy which showed chronic granulomatous inflammation with an accumulation of epithelioid macrophages, areas of fibrosis with no necrosis or microorganisms. Sputum testing with Ziehl-Neelsen stain for* Mycobacterium tuberculosis *was negative at the time. A chest X-ray (Figure 1) and CT scan of the neck (Figure 2) were done.

The patient was discharged with conservative treatment and follow-up.

**Figure 3:**
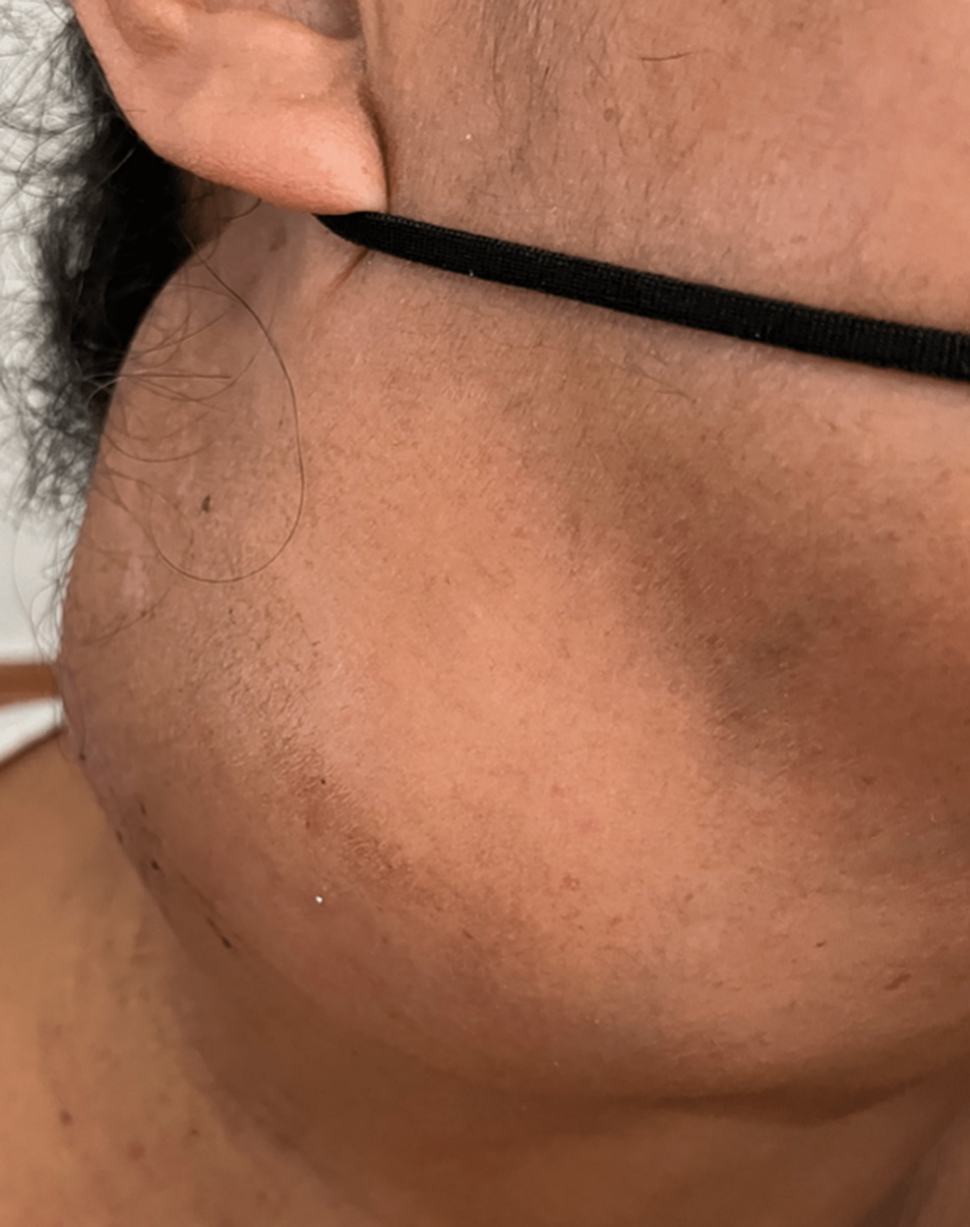
Right painless mobile cervical mass with smooth consistency showing no signs of inflammation, measuring approximately 10x10 cm.

The patient at follow-up came back two months later with persistence of swelling and a gradual increase in its size. Basic laboratory tests (CBC, urinalysis) were within normal ranges. HIV testing and VDRL were negative. A Mantoux skin test was not done. Physical examination of the neck showed a right painless mobile cervical mass with smooth consistency with no signs of inflammation measuring approximately 10x10 cm (Figure 3).

There were no cutaneous lesions, and no peripheral lymphadenopathy (inguinal, supraclavicular, axillary, popliteal), and the rest of the physical examination was normal. She underwent a biopsy which showed diffuse inflammatory infiltration with an accumulation of epithelioid macrophages and features of caseous necrosis. Diagnosis of primary tuberculous lymphadenitis was made and 2HRZE + 4HR regimen based on our local guidelines for the management of tuberculosis was started.

## Discussion

Neck masses are a very common finding in patients; they can be located below the mandible, above the clavicle, and deep into the skin. They may develop from multiple etiologies including infection, inflammation, congenital, traumatic, benign, or malignant processes [2]. The latest guidelines on the evaluation of neck masses in adults recommend that a neck mass should be considered malignant until proven otherwise, therefore further investigation should always be done [3,4].

The use of medical imaging is very important in these patients; contrast-enhanced CT or MRI will help localize and characterize the mass and identify other masses which were not detected initially as well as monitor organs that are potential sites of primary malignancy [4].

Even after imaging, a biopsy is needed for tissue diagnosis. The investigation performed depends on the institution, physician choice, and patient factors. Still, the latest guidelines recommend fine needle aspiration as the best initial test and should be the initial test for histologic evaluation [4,5]. Another option is an open biopsy which is the most definitive way of obtaining a diagnosis and is often done in the operating room [6]. In our case, the patient underwent an open biopsy because her first biopsy was inconclusive and the diagnosis was still uncertain. 

Tuberculosis has remained a major global and public health challenge. According to the World Health Organization(WHO), an estimated 1.3 million TB-related deaths occurred among human immunodeficiency virus (HIV) negative populations in 2016 [7]. In 2018, an estimated 10 million persons had incident TB, and 1.5 million TB-related deaths occurred worldwide [7]. In El Salvador tuberculosis still represents an important threat to the general population. In 2016, 3,050 cases worldwide of all forms of TB were reported, with a rate of 46.5 per 100,000 inhabitants, in 2018 the WHO reported an incidence of 70 cases per 100,000 inhabitants/year [8].

Extrapulmonary tuberculosis (EPTB) is seen in nearly 15%-20% of all cases of TB. Cervical lymph nodes are the most common site of tuberculous lymphadenopathy in 60%-90% of cases [9]. Patients with tuberculosis usually present with systemic symptoms such as night sweats, fever, diaphoresis, and weight loss. Some patients don’t present with systemic symptoms such as our patient where the diagnosis is difficult.

In our case, differential diagnoses of malignancy and sarcoidosis were considered due to the result of the first biopsy. Due to the high prevalence of tuberculosis in our country and the result of the final biopsy which showed caseous necrosis, a diagnosis was made and the patient was started with antituberculous treatment. In El Salvador, patients diagnosed for the first time with tuberculosis are divided into two phases of treatment: the intensive phase consists in taking four medications (isoniazid, pyrazinamide, ethambutol, rifampicin) for two months, then the continuation phase in which the patient takes two medications (isoniazid, rifampicin) for four months [10].

There is no clear consensus on how to access extrapulmonary tuberculosis following adequate treatment. Residual lymph nodes can be seen in 15-30% of patients and may not necessarily indicate treatment failure so close monitoring is a cornerstone of the management as well as screening close contacts [11].

## Conclusions

Neck mass can have multiple causes. While evaluating these kinds of patients an adequate physical examination and diagnostic studies should be done, and epidemiologic factors should be considered. Tuberculosis of the head and neck is still a diagnostic challenge and should be kept in mind, especially in countries with a prevalence of this disease. Prognosis is favorable in patients that are adherent to the treatment, therefore close monitoring is very important.
